# FEA Simulation of Crimping Pressure Distribution in Titanium and Teflon Stapedotomy Prostheses

**DOI:** 10.3390/ma19010065

**Published:** 2025-12-23

**Authors:** Mario Ceddia, Nicola Quaranta, Vito Pontillo, Alessandra Murri, Alessandra Pantaleo, Bartolomeo Trentadue

**Affiliations:** 1 Department of Mechanics, Mathematics and Management, Polytechnic of Bari, 70125 Bari, Italy; bartolomeo.trentadue@poliba.it; 2 Department of Translational Biomedicine and Neuroscience, University of Bari Aldo Moro, 70125 Bari, Italy; nicola.quaranta72@gmail.com (N.Q.); alessandrapantaleo18@gmail.com (A.P.); 3 UOC Otolaryngology, University AOUC Policlinico di Bari, 70125 Bari, Italy; pontillovito@gmail.com (V.P.); alessandramurri20@gmail.com (A.M.)

**Keywords:** stapedotomy, crimping force, FEA, Teflon, titanium

## Abstract

Stapedotomy is performed to restore ossicular chain sound transmission by inserting a piston prosthesis that couples the long process of the incus to the oval window, thereby addressing conductive hearing loss associated with otosclerosis. This study investigates the effects of crimping force, prosthesis material, and loop geometry on incus to optimize fixation while minimizing complications such as incudal necrosis. Finite element analyses were performed to quantify interface pressures and von Mises stresses for titanium prostheses with loop-band widths of 0.2, 0.3, and 0.5 mm under crimping forces of 300–500 mN and for polytetrafluoroethylene (PTFE) prostheses with loop outer diameters (OD) of 1.2, 1.4, and 1.8 mm. The analysis results showed that PTFE prostheses generated significantly lower interface pressures and stress compared to titanium. For PTFE prostheses, the equivalent von Mises stresses remained well below the critical threshold, with values ranging from 3.5 MPa up to peaks of approximately 43 MPa depending on the loop’s outer diameter. In contrast, titanium prostheses exhibited a marked dependency on crimping force and band width. At a force of 300 mN, stresses were modest (approximately 16–24 MPa). However, when increasing the force to 400 mN, stresses approached the critical threshold (up to approximately 53 MPa). With crimping forces of 500 mN, especially with band widths greater than 0.3 mm, stresses exceeded the cortical bone strength threshold (approximately 61–64 MPa), indicating an increased risk of mechanical overload and potential incudal necrosis. These findings highlight the importance, in a clinical context, of controlling the crimping force and selecting the material and geometry of the prosthesis to achieve secure coupling while preserving the incus’s structural integrity.

## 1. Introduction

Human hearing depends on the mechanical transmission of vibrations through the ossicular chain (the malleus, incus and stapes), which matches the impedance between the air in the external auditory canal and the cochlear fluids [1,2,3]. Morphological abnormalities or abnormal ossicular mobility can lead to conductive hearing loss [4]. The fixation of the stapes footplate at the oval window, which is characteristic of otosclerosis, increases the impedance of the transmission system. This produces an enlarged air–bone gap (BAG) on pure-tone audiometry, which is often accompanied by the Carhart notch around 2 kHz [5,6,7]. Otosclerosis affects around 1% of people in certain geographical regions and is associated with a hereditary predisposition [7].

The standard of care is stapes microsurgery, which is aimed at restoring an efficient transmission chain and reducing the BAG. In 1956, John J. Shea Jr. described the stapedectomy technique, which involves the complete removal of the footplate and the covering of the oval window with an autologous graft (vein or fascia) [8]. A cylindrical prosthesis is then placed and crimped to the long process of the incus [9]. The prosthesis conveys vibrations from the incus to the inner ear, thus restoring the system’s mechanoacoustic efficiency.

Currently, stapedotomy, comprising removal of the supra-structure and controlled micro-fenestration of the footplate (approximately 0.4–0.8 mm), followed by piston insertion, has largely replaced stapedectomy due to its lower invasiveness and comparable or superior audiological outcomes [9,10,11,12].

Despite technical advances, the optimal choice of prosthesis parameters, particularly diameter and material, remains a matter of debate. Regarding the diameter, several comparative studies report better outcomes with 0.6 mm pistons than with 0.4 mm pistons [13]. A meta-analysis by Laske et al. [13] documented a higher likelihood of achieving an air–bone gap (ABG) closure of ≤10 dB with larger-diameter pistons, along with more favorable postoperative thresholds. Similarly, Blijleven et al. [14] observed greater air-conduction gain (24 vs. 20 dB) and smaller postoperative ABG (8 vs. 9 dB) when using 0.6 mm pistons. However, other studies have not identified any clinically meaningful differences. For example, a systematic review and additional clinical studies did not confirm the superiority of one diameter over another [15,16,17,18].

In terms of materials, titanium alloys, polymeric materials such as Teflon (PTFE), and shape-memory alloys such as Nitinol (Ni–Ti) are used in clinical practice [19,20].

In a comparative study, Massey et al. [19] observed a higher rate of ABG closure ≤10 dB with Teflon pistons in stapedotomy, whereas in stapedectomy, Faramarzi et al. [20] reported no material-specific advantage, with audiometric outcomes broadly comparable between Teflon and titanium.

From a mechanical stability standpoint, Gargula et al. [21] reported a tendency towards fewer dislocations with titanium prostheses than with Teflon.

Nitinol prostheses, owing to their shape-memory effect, enable thermally induced self-crimping that is more uniform than manual tightening [22,23,24]. This feature may promote stable fixation by reducing the risk of loosening or non-uniform compression of the incus. However, the nickel content of the alloy carries a rare risk of hypersensitivity in predisposed individuals [24,25,26]. Additionally, thermal activation (e.g., laser) may result in thermal injury to the long process of the incus or periosteal necrosis if the coupling is too tight [26].

Regardless of the material used, the quality of the prosthesis–incus connection is a key determinant of favorable audiologic outcomes.

A recent review of revision stapedotomy cases found a dislocated or inadequately crimped prosthesis in 81–87% of revisions [27]. Tight fixation of the prosthesis to the incus has been shown to improve sound transmission and functional results [28]. Historically, it was hypothesized that over-crimping could induce ischemia by compressing the nutrient arterial branches of the long process, leading to bony necrosis [28,29,30]. More recent evidence, particularly the study by Imre Gerlinger et al. [31], indicates that overly tight or poorly executed crimping damages the periosteum and mucosa of the incus, triggering osteoclastic activity responsible for erosion and necrosis despite preserved blood flow. The frequency of necrosis appears lower when self-crimping prostheses (e.g., PTFE or nitinol) are used, which limit the risk of excessive compression associated with manual technique [32,33,34].

With manual crimping, particularly with titanium pistons, tightening the loop around the incus is the most critical step and relies heavily on the surgeon’s tactile feedback. Restricted access and limited visualization of the inferior surface of the incus make achieving truly circumferential contact and controlling the applied force precisely challenging. Insufficient tightening can lead to slippage and dislocation, resulting in a recurrence of conductive hearing loss, while excessive tightening can cause micro-trauma, erosion or necrosis of the long process.

Robotic systems aim to stabilize manipulation and standardize force application to mitigate these challenges. Christopher et al. [35] demonstrated that the REMS (Robotic ENT Microsurgery System) stabilizes the surgeon’s hand, attenuates physiological tremors, and enables application of lower, more reproducible crimping forces, thereby enhancing tissue safety and promoting procedural standardization.

The key biomechanical question is to define the crimping force ‘window’ that ensures long-term stability without tissue injury, considering piston design and material. This constitutes a nonlinear contact problem that lends itself well to finite element modeling. The finite element method (FEM) has been widely applied to the middle ear [36,37,38], with notable contributions from Wada, Koike and Gan to the vibro-acoustic characterization of the tympanic membrane, ossicular ligaments and the oval/round window interfaces. Such models allow the incorporation of patient-specific geometries via micro-CT, viscoelastic constitutive laws for soft tissues and anisotropic bone properties. They can also incorporate realistic boundary conditions and simulate transmission to the perilymphatic compartment via fluid–structure coupling [38,39,40,41].

When applied to the crimping problem, FEM enables the simulation of a controlled tightening load on the prosthetic loop. This allows contact pressures and stress distributions along the long process of the incus to be evaluated, as well as the mechanical response of the prosthesis–incus oval window system. To date, no systematic numerical studies have examined the relationship between crimping force, piston material and the onset of incus erosion/necrosis.

Against this background, the present study aims to quantify the crimping pressure exerted on the incus as a function of piston material (Teflon and titanium). The results may facilitate intraoperative standardization and support the development of crimping devices capable of applying controlled forces tailored to prosthesis type. This would improve the reproducibility of stapes surgery and reduce complication risk, in line with the available evidence on diameters, materials and nickel-intolerant patients.

## 2. Materials and Methods

**Figure 1:**
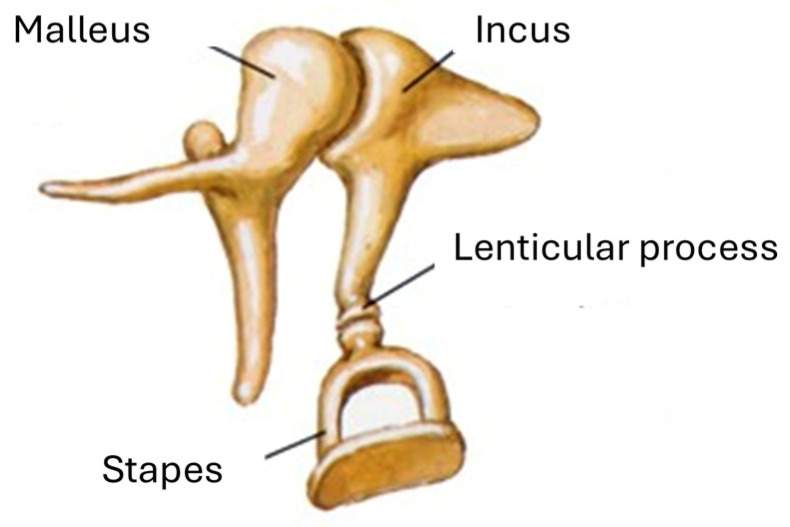
Anatomical structures of the ossicular chain.

The human ossicular chain comprises three small bones: the malleus, the incus and the stapes. The malleus is connected directly to the tympanic membrane and articulates with the body of the incus at its upper portion through the synovial incudo-malleolar joint. The long arm of the incus extends distally in the form of the lenticular process, which articulates with the head of the stapes to form the incudo-stapedial joint (see Figure 1) [28].

**Figure 2:**
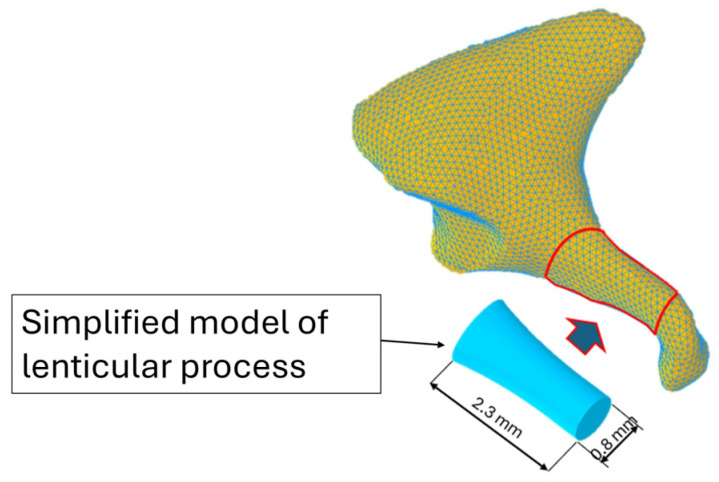
Simplified model of the lenticular process of the incus.

In accordance with the morphometric data reported by Kwok et al. [42], the lenticular process of the incus was represented as a cylinder with a 0.8 mm diameter (Figure 2). This geometric idealization was implemented using three-dimensional modeling software (Autodesk Inventor 2025), resulting in a basic model that is faithful to the anatomical parameters relevant to biomechanical analysis.

Based on the anatomical dimensions of the lenticular process of the incus, this study selected a prosthesis with an internal loop diameter of 0.8 mm and a cylindrical body diameter of 0.4 mm. The functional length of the prosthesis was set at 3.4 mm, in line with findings reported by Gentil et al. [43]. The loop was then crimped 1.3 mm from the tip of the lenticular process in line with anatomical and surgical reference guidelines.

**Figure 3:**
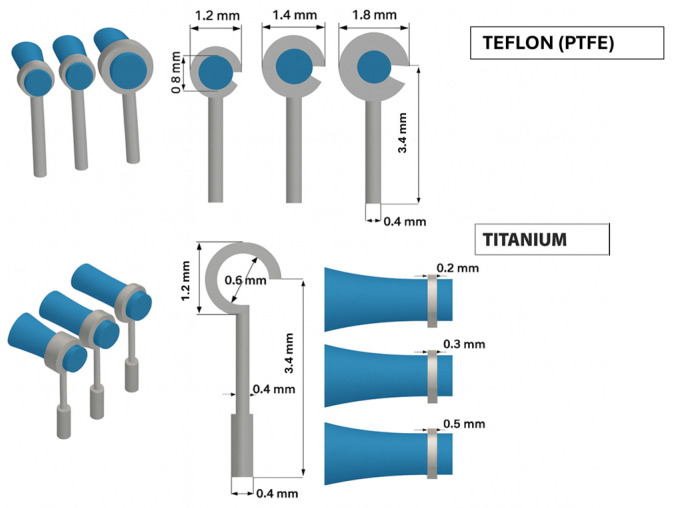
Representative models of the two prostheses in titanium and PTFE.

The analysis was conducted on two types of stapedotomy prosthesis: one made of titanium and one made of polytetrafluoroethylene (PTFE, Teflon^®^, Wilmington, DE, USA). Three configurations were considered for the titanium prosthesis, characterized by loop band widths of 0.2, 0.3 and 0.5 mm. The PTFE prosthesis was analyzed by varying the external diameter of the loop to 1.2, 1.4 and 1.8 mm (Figure 3).

The incus was modeled as cortical bone, with linear, elastic and isotropic mechanical behavior. In line with previous biomechanical studies [43,44,45], a Young’s modulus of 14 GPa and a Poisson’s ratio of 0.30 were assigned.

The prostheses were also modeled based on linear elasticity and isotropy. Specifically, a Young’s modulus of 110 GPa and a Poisson’s ratio of 0.30 were assigned to titanium, and a Young’s modulus of 0.5 GPa and a Poisson’s ratio of 0.46 to PTFE (Teflon) [41,46,47,48].

### 2.1. Evaluation of Pressure and Contact Force

To estimate the contact pressure generated by the self-crimping of a polytetrafluoroethylene (PTFE) prosthesis on the long process of the incus, the theory of cylinders under pressure was adopted, referring to Lamé’s solutions for thin cylinders [49,50]. In this approach, the prosthesis loop was modeled as an elastic ring with an internal diameter (ID) of 0.8 mm and a variable outer diameter (OD) of 1.2–1.4–1.8 mm.

The assumptions of small deformations, linear elasticity and material isotropy were made. With a fixed radial interference of *Δr* = 2 µm, the average interface pressure (*p*) was calculated using the following equation:

(1) $$p=\frac{E\left(\frac{\Delta r}{r_{m}}\right)}{\frac{r_{m}}{t}+\nu }$$

where $\left(r_{m}\right)$ represents the average radius of the loop, (*Δr*) represents the interference between the loop and the long process of the incus, (*t*) represents the thickness of the loop and (ν) represents the Poisson’s ratio of Teflon.

Equation (1) describes how to calculate contact pressure for a completely closed circular loop. However, as illustrated in Figure 3, the loop exhibits a semi-opening characterized by an opening angle (*θ*), which is required to facilitate crimping around the incus.

To account for this, the pressure calculation was adjusted to include a compliance factor, *ϕ(θ)*, which reduces the loop’s effective radial stiffness and consequently the average interface pressure:

(2) $$p_{loop}=\frac{p}{\phi \left(\theta \right)}$$

The factor ϕ(θ) was derived from ring-opening mechanics for split rings under uniform internal pressure, following the approach proposed for open thin rings. In this framework, the circumferential stiffness of an open ring decreases approximately in proportion to its angular coverage. Therefore, the ratio between the stiffness of a closed and an open ring can be expressed as:

(3) $$\phi (\theta )=\frac{2\pi }{\theta }$$

Regarding the titanium prosthesis, the contact pressure does not result from the elasticity of the material itself, but from the deformation imposed by the clamp. In this case, the loop is described as an arc with an amplitude (*θ*) and an axial width (*w*). The key quantity to analyze is the force (*F*) required to close the loop on the incus with the clamp, which is determined as follows:

(4) $$F=p\cdot A_{C}$$

where *p* = contact pressure

*Ac* = contact area, evaluated as:

(5) $$A_{C}=w\cdot L_{C}$$

*Lc* = contact length, evaluated as:

(6) $$L_{c}=\frac{\theta }{360}\cdot 2\pi r_{i}$$

where $r_{i}$ = internal radius of the loop.

### 2.2. FEA Modeling

**Figure 4:**
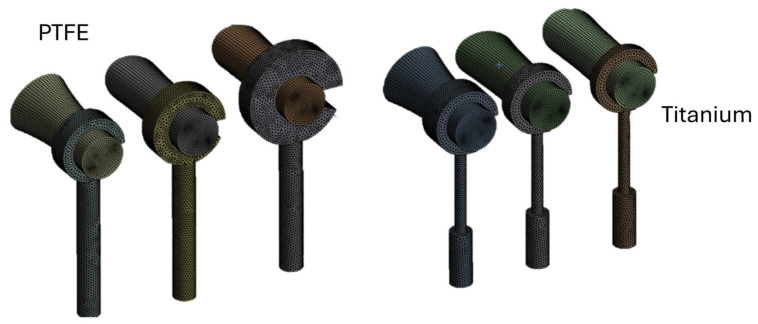
Finite element discretization using tetrahedral elements with an average size of 0.05 mm.

Following the formulation of the contact interaction (contact pressure and reaction force) in ANSYS Workbench R 2023 (FEA), a finite element model of the incus–prosthesis assembly was constructed. The domain was discretized as a three-dimensional tetrahedral mesh (average element size 0.05 mm), yielding 105,403 nodes and 24,840 elements (Figure 4).

### 2.3. Boundary Conditions

**Figure 5:**
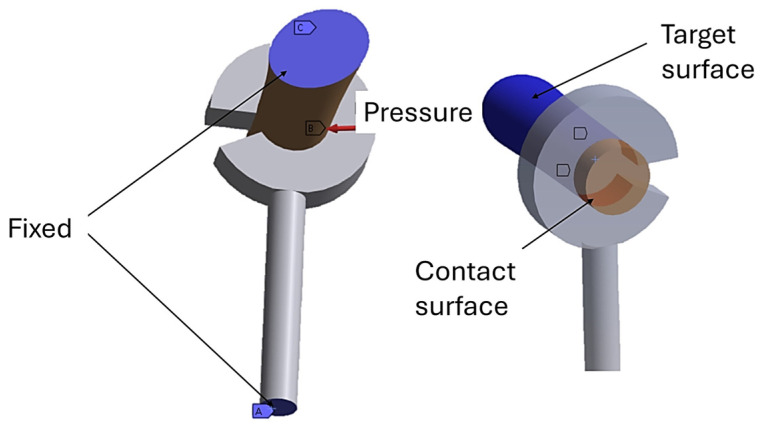
Boundary conditions for the prosthesis crimped to the incus.

To simulate the interaction between the prosthetic loop and the long process of the incus, a nonlinear contact formulation was implemented. In ANSYS, the inner surface of the loop was defined as the contact surface, whereas the cortical surface of the incus was defined as the target surface (Figure 5). A frictional contact with an Augmented Lagrangian algorithm was employed to accommodate local stick–slip behavior under geometric nonlinearity.

A friction coefficient of 0.25 was assigned to the metal–bone interface. This value lies within the range reported by tribological studies on titanium and CoCrMo surfaces sliding against cortical bone or bone analogs under simulated physiological conditions and is commonly adopted in finite element models of orthopedic implants [51,52,53,54]. For PTFE–bone contact, specific data are limited; therefore, the same value was used as a conservative assumption, which is expected to overestimate interfacial shear stresses at the PTFE–bone interface.

The two-pass (symmetric) contact option was enabled to ensure a uniform pressure distribution and automated detection of contact, and large-deformation effects (NLGEOM) were activated to capture geometry changes during crimping. This setup allows a realistic reproduction of the contact pressure and stress fields transmitted from the loop to the incus during prosthesis application.

Regarding boundary conditions, the posterior portion of the incus was fixed to suppress unwanted translations and rotations, thereby replicating its anatomical anchorage within the ossicular chain. In addition, the distal end of the prosthetic cylinder was constrained in all degrees of freedom (Figure 5).

The contact pressures reported in Table 1 for the PTFE prostheses were calculated using the classical Lamé solution for a thick-walled cylinder subjected to internal pressure. Under the assumptions of small deformations, linear elasticity and axisymmetric loading, a first expression (Equation (1)) was used to compute the average interface pressure p from the radial interference between the incus (0.8 mm diameter) and the prosthetic loop, the loop dimensions and the elastic properties of PTFE. Since this formulation strictly applies to a closed ring, the pressure was then corrected by a conformity factor that reduces the radial stiffness of the loop as a function of its opening angle θ, leading to the modified relationship in Equation (2). The resulting pressures are those listed in Table 1 and were used as boundary conditions in the FEA simulations.

These calculated pressures were then applied to the lower surface of the loop to recreate the self-crimping effect of the material on the incus.

Three levels of crimping force (300, 400, and 500 mN) were considered for the titanium prosthesis, consistent with the range reported by Christopher R. et al. [35]. In this work, forces are expressed in millinewtons (mN), where 1 Newton (N) corresponds to 1000 millinewtons (mN), i.e., 1 mN = 0.001 N.

Finite element analysis (FEA) will be used to quantify the von Mises stress generated by the pressures exerted by the PTFE (Teflon) prosthesis and the crimping forces assumed for the titanium prosthesis. These stresses will then be compared with the critical von Mises value for the incus, which is commonly indicated in the literature as the strength limit of cortical bone at 60 MPa [55].

## 3. Results

**Figure 6:**
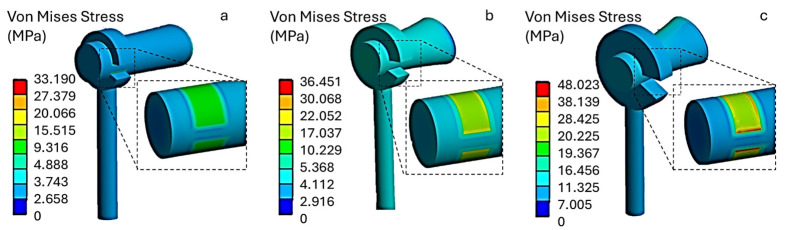
Von Mises stress distribution for a PTFE (Teflon) prosthesis, (**a**) 1.2 mm, (**b**) 1.4 mm and (**c**) 1.8 mm.

Figure 6 shows the von Mises equivalent stress distribution for a polytetrafluoroethylene (PTFE, Teflon) piston prosthesis with three different outer diameter (OD) loop configurations: (a) 1.2 mm, (b) 1.4 mm and (c) 1.8 mm.

In all analyzed conditions, stress does not exceed the critical limit of 60 MPa. Model (a) shows an average von Mises stress of 14 MPa at the prosthesis–incus contact surface, model (b) shows 19 MPa, and model (c) shows the highest values, with peaks of up to 43 MPa. This increase is due to the greater stiffness of the loop associated with the larger external diameter, which reduces local adaptability and concentrates contact forces.

For the PTFE (Teflon) prosthesis, the equivalent von Mises stresses were found to be 3.5 MPa for model (a), 9.3 MPa for model (b), and 12.3 MPa for model (c). These values do not represent a structural concern, as they are well below the tensile strength of PTFE (25 MPa). Furthermore, the estimated stress levels are also lower than the typical compressive strength limits of the material (≈30–40 MPa), confirming an adequate structural safety margin.

**Figure 7:**
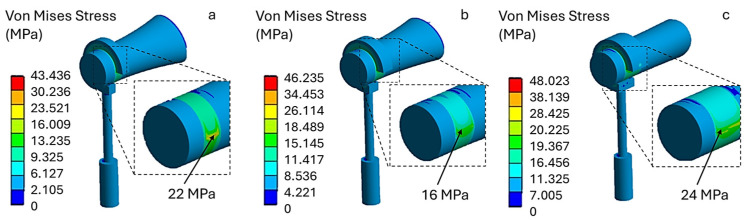
Von Mises stress distribution for a titanium prosthesis with a crimping force of 300 mN, (**a**) 0.2 mm, (**b**) 0.3 mm and (**c**) 0.5 mm.

The numerical analysis of the titanium prostheses was carried out for different loop band widths: (a) 0.2 mm, (b) 0.3 mm and (c) 0.5 mm. Figure 7 shows the von Mises stress distribution when a crimping force of 300 mN is applied.

As can be seen, the stress levels are higher than in the PTFE prosthesis. Specifically, the maximum stress on the contact surface with the incus is 22 MPa in model (a), 16 MPa in model (b), and 24 MPa in model (c). These preliminary results suggest that applying a crimping force of 300 mN does not pose a risk of incus necrosis, as the resulting stress levels remain well below the critical threshold of 60 MPa.

**Figure 8:**
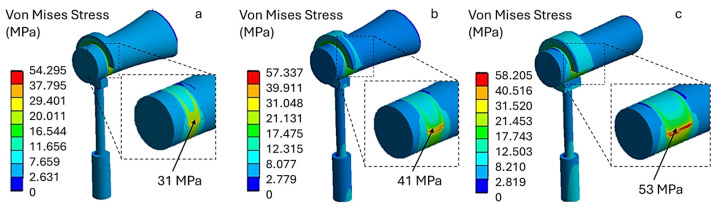
Von Mises stress distribution for a titanium prosthesis with a crimping force of 400 mN, (**a**) 31 MPa, (**b**) 41 MPa, (**c**) 53 MPa.

Increasing the crimping force to 400 mN causes the von Mises stress values to rise in all models. As shown in Figure 8, model (a) has a stress value of 31 MPa. This value increases with the width of the loop band, reaching 53 MPa in model (c). Therefore, under this loading configuration, using a wider loop band (model c) leads to higher stress, which may approach the critical stress threshold tolerated by the incus.

**Figure 9:**
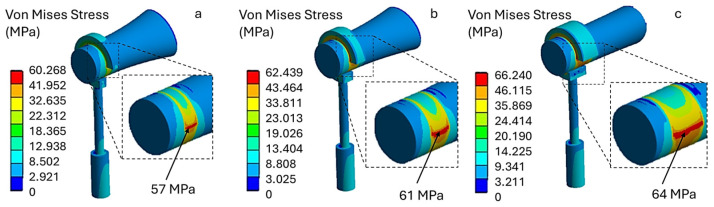
Von Mises stress distribution for a titanium prosthesis with a crimping force of 500 mN, (**a**) 57 MPa, (**b**) 61 MPa, (**c**) 64 MPa.

Finally, applying a crimping force of 500 mN results in stress values approaching the mechanical strength of the incus. As shown in Figure 9, model (a) exhibits a maximum stress of 57 MPa, model (b) exhibits a maximum stress of 61 MPa, and model (c) exhibits a maximum stress of 64 MPa. Therefore, the latter two models produce stress levels on the incus exceeding 60 MPa. These configurations may pose a risk of incus necrosis when high crimping forces are applied.

The results obtained showed that PTFE (Teflon) prostheses caused lower stress values on the incus than titanium ones did. Additionally, due to their self-crimping behavior, contact pressure was observed to increase with outer diameter (OD) of the loop.

By contrast, titanium prostheses generate higher stress levels on the incus, depending on the applied crimping force and loop band width. Notably, crimping forces exceeding 400 mN produced stress levels approaching the critical threshold associated with incus necrosis.

It should be noted that, in the titanium 500 mN condition (Figure 9), the nodes exhibiting peak von Mises stresses are located within the compressed cortical region beneath the prosthetic loop. Post-processing of principal stresses confirmed that these peaks arise from a predominantly compressive normal stress state, with superimposed deviatoric (shear) components. On the opposite side of the incus, tensile stresses are lower and associated with markedly smaller von Mises values.

From a clinical standpoint, these findings emphasize the importance of carefully controlling the crimping force during implantation. The numerical results suggest that, to minimize the risk of mechanical overloading and potential necrosis of the incus when a titanium piston prosthesis is used, crimping forces above 300 mN and loop band widths greater than 0.3 mm should be avoided.

## 4. Discussion

Stapes surgery is a highly effective treatment for otosclerosis. The transition to small-fenestra stapedotomy has generally improved audiological outcomes and reduced complications compared with traditional stapedectomy, although some historical series report broadly comparable results [10,56,57,58]. For example, Al-Husban et al. [9] documented an air–bone gap (ABG) closure ≤10 dB in 88% of cases after stapedotomy versus 64% after stapedectomy (*p* < 0.05), with fewer complications in the stapedotomy group. In contrast, Quaranta et al. [59] observed postoperative hearing results that were comparable between stapedotomy and partial stapedectomy within the speech-frequency range.

Despite these favorable outcomes, a conductive hearing loss of >20 dB may occur early or late and necessitate revision surgery. In the review by Sakano et al. [60], the most common indications for re-intervention were prosthesis displacement/malfunction, incus necrosis, re-obliteration of the oval window, malleus/incus ankylosis, and granuloma. Prosthesis malfunction emerged as the leading cause (42.6%), followed by incus necrosis (22%).

The quality of loop fixation on the long process of the incus (LPI) is critical. Historically, necrosis was attributed to excessive tightening [61], whereas current evidence indicates that under-crimping induces micromotion and friction, promoting erosion of the lenticular process. In a study conducted by Huber et al. [26], inadequate fixation produced sound-transfer losses up to 28 dB, while a stable crimp with at least two opposing contact points limited losses to ≤7 dB, approximating the physiological performance of the incudostapedial joint.

Both design and materials directly affect contact pressures and mechanical stability. Band-type loops distribute forces more uniformly than wire loops, thereby reducing peak pressures on the LPI, although excessive band width may misalign the piston and degrade performance [62,63]. Among materials, shape-memory alloys (NiTi) enable thermally induced self-crimping of the loop, standardizing fixation and reducing dependence on manual skill, as shown by Harris et al. [64]. However, thermal activation (CO_2_/KTP laser or micro-bipolar) carries a risk of local thermal injury, and the nickel component raises concerns about hypersensitivity. A recent systematic review by Ahena et al. [65] reported overall complication rates >6% for heat-crimped devices compared with manually crimped prostheses, while Brown et al. [22] noted ~17% nickel patch-test positivity in the general population.

As an alternative, all-PTFE (Teflon) prostheses use a pre-shaped fluoroplastic loop: the surgeon opens, positions, and releases the loop, which elastically recovers to embrace the LPI. Fixation is achieved through circumferential tension and friction over a relatively broad contact area, without plastic crimping or heat. Audiological outcomes are comparable to those of titanium and Nitinol solutions, with the added advantages of no thermal activation, no nickel, simpler instrumentation, and lower cost [18,66,67].

In the present numerical study, we compared a titanium prosthesis (band width 0.2–0.5 mm) with a PTFE prosthesis (loop outer diameter 1.2–1.8 mm), focusing on crimping force/pressure and stresses at the incus–prosthesis interface. PTFE configurations generated lower stresses that remained <60 MPa in all cases; increasing loop outer diameter stiffened the ring and raised local peaks (up to ~43 MPa), yet within a safe margin. Titanium prostheses exhibited a strong dependence on crimping force: at 300 mN, stresses were modest (~16–24 MPa); at 400 mN, they approached the critical threshold (up to ~53 MPa); and at 500 mN, the threshold was exceeded (~61–64 MPa), particularly with band width ≥0.3 mm, indicating a potentially hazardous configuration for the incus.

The clinical translation of these findings is consistent with the work of Christopher et al. [35], who showed that the REMS robotic system reduced peak crimping forces from approximately 469 mN during manual crimping to about 273 mN. Particularly for load-sensitive titanium pistons, strategies that limit the applied tightening force (robotic assistance or standardized force-control protocols) are likely to keep incudal stress below critical thresholds and thereby reduce the risk of complications. Overall, material selection and control of crimping force are key to optimizing loop stability and the biomechanical safety of the incus–prosthesis interface.

The crimping forces used in this study should be interpreted considering experimental “admissible force” data at the long process of the incus. Lauxmann et al. [68] identified three force ranges at the incudomalleolar joint: micro-rupture at approximately 406–568 mN, rupture at 695–894 mN, and short-term maximum forces approaching 0.9–1.3 N, depending on loading direction.

In a 3D-printed temporal bone model, junior surgeons applied peak forces of 433 ± 334 mN at the incus and 565 ± 233 mN at the stapes during crimping, whereas senior surgeons applied 182 ± 169 mN and 66 ± 49 mN, respectively [69]. Taken together, these data indicate that crimping forces in clinical practice generally fall within a broad 200–500 mN window, with less experienced surgeons more likely to reach the upper end of this range.

Within this framework, the 300–500 mN loads adopted in the present finite element model represent the upper segment of clinically relevant forces. Values around 300 mN are compatible with secure coupling while remaining below the micro-rupture threshold, whereas forces in the 400–500 mN range approach the onset of micro-damage. Our simulations show that titanium band-type pistons become load-sensitive in this upper range, while PTFE loops keep incudal von Mises stresses below the 60 MPa cortical-bone limit even when crimping forces approach 500 mN. These findings support the combined use of force-limiting strategies (e.g., robotic assistance, standardized force-control protocols) and low-stiffness, self-crimping materials to buffer unavoidable surgeon-dependent variability.

### Limitations

Like all finite element analyses of the middle ear, this study relies on geometric and mechanical simplifications to obtain a tractable and numerically robust model. The complex ossicular anatomy and surrounding soft tissues are only partially represented, and the long process of the incus is modeled as a smooth cylinder derived from morphometric data.

In vivo, this region exhibits curvature, surface roughness and inter-individual variability. However, in the crimping zone, the local interface can be reasonably approximated as quasi-cylindrical. This means that contact stresses mainly depend on crimping force, loop stiffness and effective contact area. To mimic a quasi-static crimping maneuver, the distal tip of the prosthesis was fully constrained and the incus stabilized, with the finite compliance of the ligaments and tympanic membrane being neglected. While these boundary conditions likely stiffen the response and lead to conservative (higher) stress estimates, they are applied identically to titanium and PTFE loops.

Another limitation is the constitutive description of the materials. Titanium, PTFE and cortical bone were modeled as linear elastic and isotropic under small deformations. However, this approach neglects bone anisotropy and PTFE viscoelasticity, which may affect the absolute stress values. Nevertheless, the relative trends between materials are expected to remain robust under the quasi-static, short-duration loading conditions considered. Future studies integrating local experimental measurements or more advanced material models (e.g., anisotropic or viscoelastic formulations) could enable more detailed quantitative validation of the stress fields at the incus–prothesis interface.

## 5. Conclusions

Finite element analysis showed that PTFE pistons produce lower, more uniformly distributed contact stresses on the incus than titanium models, remaining within safety margins. PTFE prostheses, with loop outer diameters ranging from 1.2 mm to 1.8 mm, generated stress below 60 MPa, with local peaks up to ~43 MPa. Titanium pistons, however, exhibited marked sensitivity to crimping force and band width (0.2–0.5 mm). At a crimping force of 300 mN, stresses were modest (~16–24 MPa). However, at 400 mN, they approached the critical threshold (~53 MPa), and at 500 mN, the threshold was exceeded (~61–64 MPa), particularly with band widths ≥0.3 mm, indicating a potentially hazardous configuration. Clinically, smaller loop diameters should be selected for PTFE pistons. For titanium, force-controlled implantation using calibrated gauges or robotic assistance is crucial to prevent over-compression and incus necrosis. Crimping forces above 300 mN and loop band widths greater than 0.3 mm should be avoided for titanium. PTFE offers a practical option for patients with fragile bones or nickel sensitivity, while titanium is appropriate when precise force control is reliably achieved.

## Figures and Tables

**Table 1:** Pressure applied by the Teflon prosthesis on the incus as a function of the loop outer diameter (OD).

OD (mm)	$p_{loop}$ (MPa)
1.2	0.405
1.4	0.476
1.8	0.524

## Data Availability

The original contributions presented in this study are included in the article. Further inquiries can be directed to the corresponding author.
